# Supplementary material for: The interactome of intact mitochondria by cross-linking mass spectrometry provides evidence for coexisting respiratory supercomplexes
Source: Mol Cell Proteomics. 2017 Dec 8;17(2):216–32. doi: 10.1074/mcp.RA117.000470 (PMC5795388; doi:10.1074/mcp.RA117.000470)
Supplement: Supplemental Data [file supp_RA117.000470_133922_0_supp_23978_fzffwf.zip › spectra_annotation/mito_spectra_annotation/281-1-2-1-5-1.pdf]

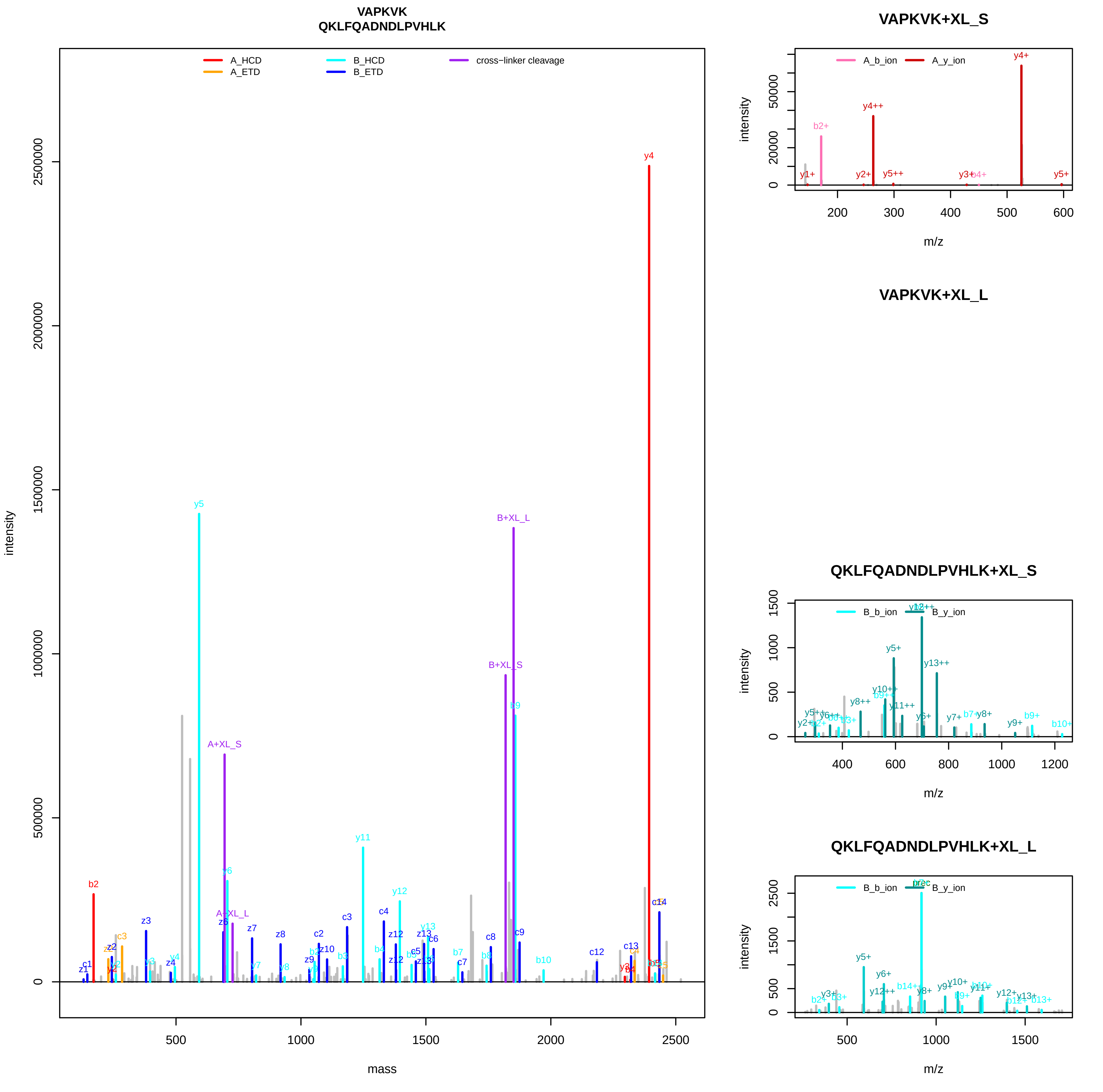
VAPKVK
QKLFQADNDLPVHLK
A_HCD
A_ETD
B_HCD
B_ETD
cross−linker cleavage
intensity
mass
0
500000
1000000
1500000
2000000
2500000
500
1000
1500
2000
2500
y4
y5
B+XL_L
B+XL_S
b9
A+XL_S
y11
y6
A+XL_L
b2
y12
c14
c3
c4
z3
z6
z7
z8
c2
z10
z12
y13
z13
c6
c8
c9
c12
c13
c5
c7
b7
b8
b10
z4
y4
z2
c3
z1
c1
z9
VAPKVK+XL_S
A_b_ion
A_y_ion
intensity
m/z
0
20000
50000
200
300
400
500
600
y4+
y4++
b2+
y1+
y2+
y5++
y3+
b4+
y5+
VAPKVK+XL_L
QKLFQADNDLPVHLK+XL_S
B_b_ion
B_y_ion
intensity
m/z
0
500
1000
1500
400
600
800
1000
1200
y12++
y5+
y13++
y10++
y8++
y11++
y7+
y8+
y9+
b9+
b10+
QKLFQADNDLPVHLK+XL_L
B_b_ion
B_y_ion
intensity
m/z
0
500
1500
2500
500
1000
1500
prec
y5+
y6+
y12++
b14+
y8+
y9+
y10+
y11+
y12+
y13+
b13+
y3+
b2+
b3+
